# Differential Impact of Bacterial Infection on Preterm Birth in Singleton and Twin Pregnancies and Its Association with Cervical Cerclage or Arabin Pessary: A Retrospective Cohort Study

**DOI:** 10.3390/jcm15166254

**Published:** 2026-08-13

**Authors:** Triada Charmanidou, Christina Pagkaki, Nektaria Kritsotaki, Efthymios Oikonimou, Vasiliki Kourti, Nikolaos Tsikouras, Anastasia Bothou, Nikolaos Machairiotis, Roland Csorba, Nikoletta Koutlaki, Panagiotis Tsikouras

**Affiliations:** 1 Department of Obstetrics and Gynecology, Democritus University of Thrace, 68100 Alexandroupolis, Greece; rxarma@yahoo.gr (T.C.); christinapagkaki@gmail.com (C.P.); nektaria.97@hotmail.com (N.K.); eftoikonomou@outlook.com (E.O.);; 2 Department of Midwifery, School of Health Sciences, University of West Attica (UNIWA), 12243 Egaleo, Greece; 3 Department of Obstetrics and Gynecology, Attikon, 12462 Athens, Greece; nikolaosmachairiotis@gmail.com; 4 Department of Obstetrics and Gynecology, University Clinicum, 45147 Essen, Germany; drcsorbaroland@gmail.com

**Keywords:** preterm birth, bacterial infection, singleton pregnancy, twin pregnancy, cervical pessary, cervical cerclage, prematurity

## Abstract

**Background:** Bacterial infection’s differential impact in singleton versus twin pregnancies and the modifying role of cervical interventions remain unclear. The objective is to investigate the association between bacterial infection and preterm birth in singleton and twin pregnancies and to evaluate whether cervical intervention modifies this relationship. **Methods:** A retrospective cohort study was conducted at a tertiary referral maternity center. Medical records of 146 pregnancies managed between December 2019 and December 2024 were reviewed, including 70 singleton and 76 twin pregnancies. Bacterial infection was defined as the presence of at least one positive microbiological culture during pregnancy. Associations between bacterial infection and gestational age at delivery were assessed using chi-square tests and binary logistic regression analyses. Additional models evaluated the effects of cervical intervention and interactions between bacterial infection and gestational type. **Results:** Among singleton pregnancies, bacterial infection was significantly associated with earlier preterm birth in the unadjusted analysis. Positive cultures were identified in 66.7% of women delivering between 28 and 33 weeks compared with 20.6% of those delivering between 34 and 37 weeks (χ^2^ = 15.047, *p* < 0.001). However, bacterial infection was not independently associated with preterm birth in the regression model including intervention type and the infection-by-intervention interaction (OR = 0.375, 95% CI 0.049–2.887, *p* = 0.346). In twin pregnancies, no significant association was observed, with similar rates of positive cultures across gestational age categories (45.5% vs. 40.6%; χ^2^ = 0.176, *p* = 0.675). Cervical intervention did not significantly modify the relationship between bacterial infection and preterm birth in either gestational group. In the combined model, a significant interaction was identified between gestational type and bacterial infection (OR = 0.158, 95% CI 0.038–0.655, *p* = 0.011). **Conclusions:** In this retrospective cohort, bacterial infection was associated with earlier preterm birth in singleton pregnancies in unadjusted analyses, but this association was not confirmed in the regression model. No significant association was observed in twin pregnancies. The interaction between gestational type and bacterial infection suggests that this relationship may differ according to pregnancy type, while cervical interventions did not appear to modify it. These findings should be considered exploratory and require confirmation in larger prospective studies.

## 1. Introduction

Preterm birth remains one of the leading causes of neonatal morbidity and mortality worldwide and represents a major public health challenge despite substantial advances in obstetric and neonatal care. Approximately 15 million neonates are born preterm each year, and complications related to prematurity account for a significant proportion of neonatal deaths, as well as long-term neurodevelopmental impairment worldwide [1]. In addition to its impact on neonatal survival, preterm birth is associated with increased risks of respiratory distress syndrome, necrotizing enterocolitis, cerebral palsy, intraventricular hemorrhage, and long-term cognitive and behavioral disorders, imposing substantial healthcare and socioeconomic burdens [1].

Preterm birth is increasingly viewed as a multifactorial syndrome rather than a single disease entity. Several mechanisms have been implicated in its pathogenesis, including infection, uteroplacental ischemia, cervical insufficiency, decidual hemorrhage, genetic susceptibility, and uterine overdistension [2]. These mechanisms often converge on shared biological pathways leading to cervical remodeling, membrane rupture, increased uterine contractility, and premature delivery. Among these pathways, infection remains one of the most important contributors to spontaneous preterm labor, particularly at earlier gestational ages [3].

The association between microbial infection and preterm birth has been extensively investigated. Ascending infection from the lower genital tract is considered the most common route through which microorganisms gain access to the fetal membranes and amniotic cavity. Once microorganisms reach the intrauterine environment, activation of maternal and fetal immune responses results in increased production of inflammatory cytokines, chemokines, prostaglandins, and matrix metalloproteinases [3,4]. These mediators promote cervical ripening, degradation of the extracellular matrix, weakening of the fetal membranes, and uterine contractions, thereby facilitating the onset of preterm labor and delivery [4].

Recent evidence has further highlighted the importance of inflammatory activation as a key biological mechanism linking infection to preterm birth. Both microbial invasion and sterile inflammatory responses may activate similar molecular pathways involving interleukin-1β, interleukin-6, tumor necrosis factor-α, and other pro-inflammatory mediators that contribute to the initiation of labor [4,5].

The maternal vaginal microbiome has also emerged as an important factor influencing pregnancy outcomes. Alterations in vaginal microbial composition have been associated with an increased risk of spontaneous preterm birth [6]. Studies have demonstrated that disruption of the normal Lactobacillus-dominant vaginal microbiota may predispose women to ascending infection and subsequent inflammatory responses within the intrauterine environment [6]. These findings further support the complex interaction between microbial colonization, host immune responses, and the risk of prematurity.

Although the role of infection has been extensively investigated in singleton pregnancies, considerably less is known about its contribution to preterm birth in twin gestations. Twin pregnancies carry a substantially higher baseline risk of prematurity, with more than half resulting in delivery before 37 weeks of gestation [7]. Prematurity remains the principal determinant of neonatal morbidity and mortality in twins and contributes significantly to adverse perinatal outcomes [7].

The biological mechanisms leading to preterm birth in twin pregnancies may differ from those observed in singleton gestations. In addition to infection-related pathways, uterine overdistension, increased placental mass, altered endocrine signaling, and accelerated cervical shortening have been proposed as important contributors to premature delivery in twin pregnancies [2,7]. These factors may reduce the relative impact of infection-mediated mechanisms and potentially explain differences in the association between bacterial infection and prematurity across gestational types.

Because cervical shortening and cervical insufficiency play critical roles in the development of spontaneous preterm birth, several preventive interventions have been proposed for women at increased risk. Cervical pessary placement and cervical cerclage are among the most frequently used strategies aimed at reducing preterm birth through mechanical support of the cervix [8,9]. Cervical pessaries are thought to alter cervical geometry and redistribute uterine pressure away from the internal cervical os, whereas cerclage reinforces the cervix through surgical placement of a suture [8,9]. Despite widespread clinical use, the effectiveness of these interventions remains controversial in certain populations.

Persistent infection-related inflammatory activity may promote cervical remodeling and membrane weakening despite mechanical support, potentially limiting the protective effect of pessaries or cerclage in some patients [10]. Determining whether bacterial infection alters the relationship between cervical interventions and preterm birth is therefore clinically important.

Although the contribution of infection to spontaneous preterm birth has been extensively investigated, comparatively fewer studies have directly evaluated whether this association differs between singleton and twin pregnancies while simultaneously considering cervical interventions. Clarifying these relationships may improve our understanding of the heterogeneous mechanisms underlying preterm birth in different gestational settings.

Therefore, the primary aim of the present study was to investigate the association between bacterial infection and preterm birth in singleton and twin pregnancies. A secondary objective was to evaluate whether cervical intervention modifies these associations and to determine whether the relationship between bacterial infection and prematurity differs according to gestational type.

## 2. Materials and Methods

### 2.1. Study Design and Setting

We conducted a retrospective cohort study at the University Hospital of Alexandroupolis, a tertiary referral center for maternal–fetal medicine, including pregnancies managed between December 2019 and December 2024. Ethical approval was obtained from the Institutional Review Board of the University Hospital of Alexandroupolis (Approval Number RF 43760/15.10.2021). Due to the retrospective nature of the study and the use of anonymized clinical data, the requirement for individual informed consent was waived.

A total of 146 pregnancies met the eligibility criteria and were included in the final analysis, comprising 70 singleton pregnancies and 76 twin pregnancies. All data were anonymized prior to statistical analysis.

### 2.2. Study Population

Medical records of pregnant women managed during the study period were retrospectively reviewed. Eligible pregnancies were required to have documented microbiological culture results, intervention status, and gestational age at delivery.

### 2.3. Inclusion Criteria

1. Singleton or twin pregnancy.
2. Available microbiological culture results during pregnancy.
3. Known intervention category.
4. Documented gestational age at delivery.
5. Complete clinical records for the variables included in the analysis.

### 2.4. Exclusion Criteria

1. Major fetal structural or chromosomal abnormalities.
2. Missing microbiological culture data.
3. Missing intervention information.
4. Missing gestational age at delivery.
5. Incomplete medical records precluding outcome assessment.

### 2.5. Missing Data

Because inclusion required complete documentation of all study variables, the final analytic cohort represented a complete-case sample. Consequently, the possibility of selection bias due to excluded incomplete records cannot be excluded. This approach was considered appropriate given the retrospective design of the study and the relatively limited proportion of incomplete records.

### 2.6. Exposure Variable: Bacterial Infection

Bacterial infection was defined as the presence of at least one positive microbiological culture documented during pregnancy. Pregnancies with positive culture findings were classified as infected, whereas pregnancies with negative culture results were classified as non-infected. Because of the retrospective design, microbiological findings were analyzed as a composite exposure irrespective of the sampling site, bacterial species, or timing during pregnancy, as these data were not consistently available for all patients.

### 2.7. Intervention Classification

Interventions were categorized according to the clinical management strategy employed:

1. Cervical pessary.
2. Cervical cerclage.
3. Conservative management (expectant management).

For interaction analyses, intervention categories were combined when subgroup sample sizes were insufficient to support stable logistic regression models. In singleton pregnancies, intervention was classified as pessary versus other interventions. In twin pregnancies, intervention was classified as pessary versus other interventions.

Selection of intervention was based on routine clinical decision-making by the treating physician and reflected factors including cervical findings, obstetric history, gestational age, and perceived risk of preterm birth. Because treatment allocation was not randomized, the study is susceptible to confounding by indication. Similarly, microbiological cultures were obtained according to routine clinical practice rather than a predefined research protocol, introducing the possibility that women with symptoms or increased clinical concern were more likely to undergo microbiological testing. This limitation should be considered when interpreting the observed associations.

### 2.8. Outcome Definition

The primary outcome was preterm birth.

For singleton pregnancies, gestational age at delivery was categorized as:

- 28–33 weeks.
- 34–37 weeks.

For twin pregnancies, gestational age at delivery was categorized as:

- 29–33 weeks.
- 34–36 weeks.

For analyses comparing singleton and twin pregnancies, gestational age at delivery was further dichotomized as:

- ≤33 weeks.
- >33 weeks.

The gestational-age categories differed between singleton and twin pregnancies because the observed ranges of gestational age at delivery were not identical in the two cohorts. For the combined analysis, gestational age was dichotomized as ≤33 versus >33 weeks to preserve consistency with the gestational-age categories used in the separate singleton and twin analyses.

Gestational age was determined using first-trimester ultrasonography whenever available or by the date of the last menstrual period when early ultrasound dating was unavailable.

### 2.9. Statistical Analysis

Categorical variables are summarized using frequencies and percentages. Associations between bacterial infection and preterm birth were evaluated using Pearson’s chi-square test. Fisher’s exact test was used when appropriate.

Binary logistic regression analyses were subsequently performed to investigate:

1. The association between bacterial infection and preterm birth in singleton pregnancies.
2. The association between bacterial infection and preterm birth in twin pregnancies.
3. Whether intervention modified the association between bacterial infection and preterm birth in singleton pregnancies.
4. Whether intervention modified the association between bacterial infection and preterm birth in twin pregnancies.
5. Whether the association between bacterial infection and preterm birth differed between singleton and twin pregnancies.

The regression models were limited to variables consistently available across the cohort. Maternal age, parity, previous preterm birth, and antibiotic treatment were not included because complete data were not available for all participants.

Odds ratios (ORs) and corresponding 95% confidence intervals (95% CIs) were calculated. Model fit was assessed using omnibus tests of model coefficients, classification accuracy, and Nagelkerke’s R^2^. Statistical significance was defined as a two-sided *p*-value < 0.05.

All statistical analyses were performed using IBM SPSS Statistics, version 28.0 (IBM Corp., Armonk, NY, USA). Given the exploratory nature of this retrospective study, no formal adjustment for multiple comparisons was applied. Accordingly, the findings, particularly those derived from interaction analyses, should be interpreted as hypothesis-generating.

## 3. Results

### 3.1. Study Population

A total of 146 pregnancies were included in the study, comprising 70 singleton pregnancies and 76 twin pregnancies. Among singleton pregnancies, 31 women (44.3%) had a documented positive bacterial culture and 39 (55.7%) had negative cultures. Among twin pregnancies, 33 women (43.4%) had a positive bacterial culture and 43 (56.6%) had negative cultures (Table 1).

### 3.2. Association Between Bacterial Infection and Preterm Birth in Singleton Pregnancies

Among singleton pregnancies, bacterial infection was significantly associated with earlier preterm birth. Of the 34 women who delivered between 34 and 37 weeks of gestation, 7 (20.6%) had a positive bacterial culture, whereas 27 (79.4%) had negative cultures. In contrast, among the 36 women who delivered between 28 and 33 weeks, 24 (66.7%) had a positive bacterial culture and 12 (33.3%) had negative cultures.

Pearson’s chi-square analysis demonstrated a statistically significant association between bacterial infection and gestational age at delivery (χ^2^ = 15.047, *p* < 0.001), indicating that bacterial infection was more frequently observed among women delivering at earlier gestational ages (Table 2).

### 3.3. Association Between Bacterial Infection and Preterm Birth in Twin Pregnancies

**Figure 1:**
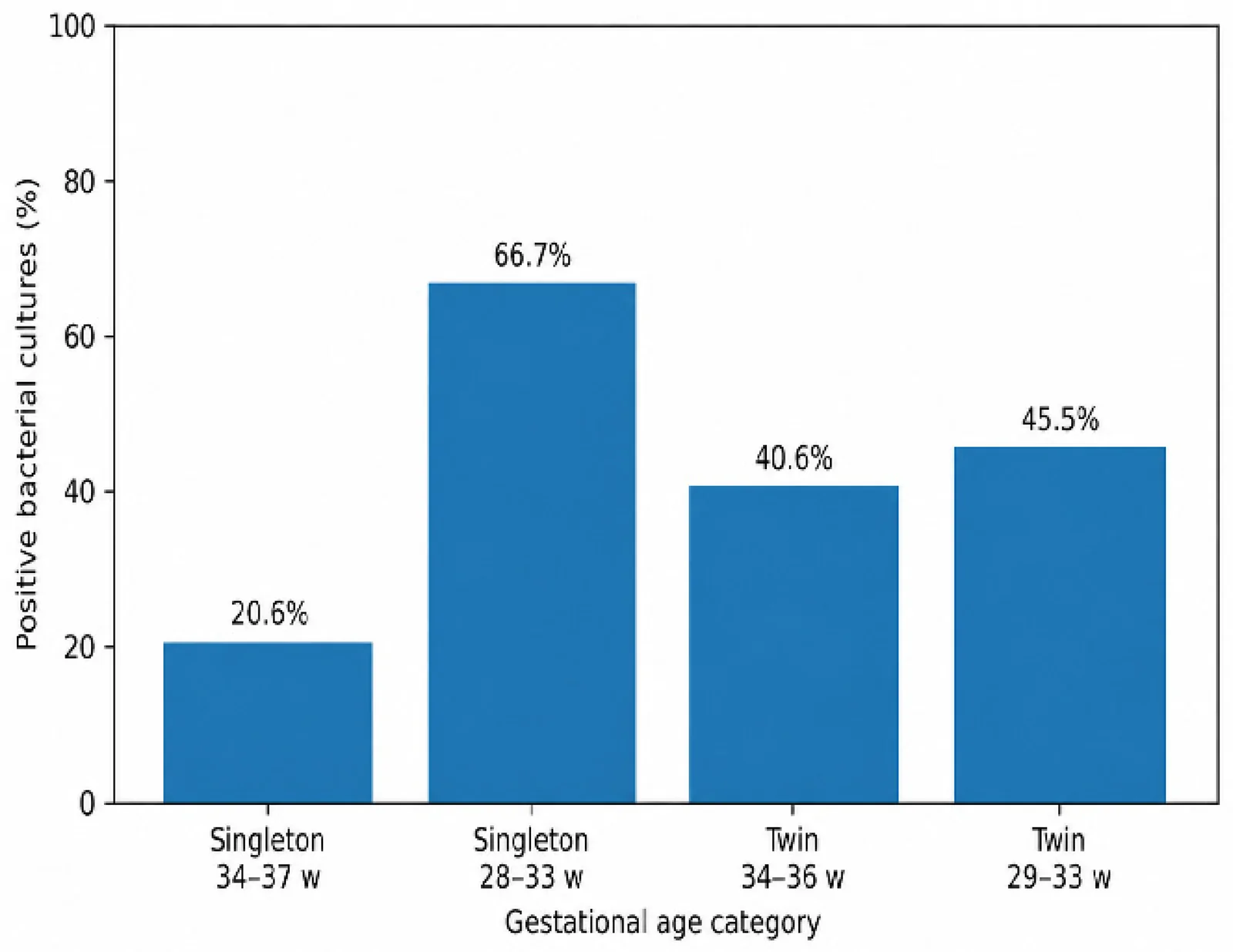
Prevalence of bacterial infection according to gestational age category in singleton and twin pregnancies. The proportion of pregnancies with positive bacterial cultures was substantially higher among singleton pregnancies delivering at 28–33 weeks compared with those delivering at 34–37 weeks (66.7% vs. 20.6%). In contrast, the prevalence of bacterial infection was similar between gestational age categories in twin pregnancies (45.5% vs. 40.6%).

Among twin pregnancies, bacterial infection was not significantly associated with gestational age at delivery. Positive bacterial cultures were documented in 13 of 32 pregnancies (40.6%) delivering between 34 and 36 weeks and in 20 of 44 pregnancies (45.5%) delivering between 29 and 33 weeks (Figure 1).

No statistically significant association was observed between bacterial infection and prematurity in twin pregnancies (χ^2^ = 0.176, *p* = 0.675) (Table 3).

### 3.4. Effect of Intervention on the Association Between Bacterial Infection and Preterm Birth

Binary logistic regression was performed to evaluate whether intervention type modified the association between bacterial infection and preterm birth (Table 4).

In singleton pregnancies, the overall model was statistically significant (Omnibus χ^2^ = 17.079, *p* = 0.001), explaining 28.9% of the variance in preterm birth according to the Nagelkerke R^2^ statistic. However, neither bacterial infection status (OR 0.38, 95% CI 0.05–2.89, *p* = 0.346), intervention type (OR 2.25, 95% CI 0.39–13.07, *p* = 0.366), nor the interaction between bacterial infection and intervention (OR 0.23, 95% CI 0.02–2.64, *p* = 0.239) independently predicted preterm birth.

Similarly, in twin pregnancies, neither bacterial infection (OR 0.71, 95% CI 0.14–3.62, *p* = 0.681), intervention type (OR 0.25, 95% CI 0.05–1.17, *p* = 0.078), nor the interaction term (OR 1.23, 95% CI 0.16–9.39, *p* = 0.841) reached statistical significance.

These findings suggest that intervention type did not significantly modify the relationship between bacterial infection and preterm birth in either singleton or twin pregnancies.

### 3.5. Comparison Between Singleton and Twin Pregnancies

To investigate whether the relationship between bacterial infection and preterm birth differed according to gestational type, a binary logistic regression model including pregnancy type, bacterial infection status, and their interaction was performed.

The overall model was statistically significant (Omnibus χ^2^ = 16.512, *p* = 0.001) and explained 14.3% of the variance in preterm birth (Nagelkerke R^2^ = 0.143). The interaction between pregnancy type and bacterial infection was statistically significant (OR 0.16, 95% CI 0.04–0.66, *p* = 0.011), whereas neither pregnancy type alone (OR 2.23, 95% CI 0.75–6.65, *p* = 0.151) nor bacterial infection status alone (OR 0.82, 95% CI 0.33–2.06, *p* = 0.675) reached statistical significance.

These findings indicate that the association between bacterial infection and preterm birth differs significantly between singleton and twin pregnancies. Specifically, bacterial infection was strongly associated with earlier preterm birth in singleton pregnancies, whereas no significant association was observed in twin pregnancies (Table 4).

The significant association observed in the chi-square analysis but not in the multivariable logistic regression model may reflect the relatively small sample size and the instability of regression estimates when interaction terms are included. Consequently, the lack of statistical significance in the multivariable model should be interpreted cautiously rather than as definitive evidence of the absence of an association.

## 4. Discussion

### 4.1. Principal Findings

In this retrospective cohort study of 146 singleton and twin pregnancies, bacterial infection was significantly associated with earlier preterm birth in singleton pregnancies but not in twin pregnancies. Positive bacterial cultures were markedly more frequent among singleton pregnancies delivering between 28 and 33 gestational weeks compared with those delivering between 34 and 37 weeks, whereas no significant differences were observed among twin pregnancies. Furthermore, cervical intervention type did not significantly modify the relationship between bacterial infection and preterm birth in either gestational group. Importantly, the significant interaction between bacterial infection and gestational type suggests that the contribution of infection-related pathways to preterm birth may differ between singleton and twin pregnancies. However, because multiple regression models were evaluated without formal correction for multiple comparisons, this interaction should be interpreted cautiously and considered exploratory. Although the association was statistically significant in the unadjusted analyses, it was not confirmed in the logistic regression model.

### 4.2. Interpretation of Findings

The association observed between bacterial infection and earlier preterm birth in singleton pregnancies is biologically plausible and consistent with current understanding of spontaneous preterm labor. Preterm birth is increasingly recognized as a syndrome with multiple etiologies that converge on common biological pathways leading to cervical remodeling, membrane rupture, and uterine activation. Infection represents one of the best-established mechanisms contributing to spontaneous preterm labor, particularly at earlier gestational ages [11]. Microbial invasion of the reproductive tract may trigger maternal and fetal inflammatory responses characterized by increased production of cytokines, chemokines, prostaglandins, and matrix metalloproteinases. These mediators contribute to cervical ripening, extracellular matrix degradation, membrane weakening, and the initiation of uterine contractions [3,12].

Our findings are also consistent with evidence linking alterations of the vaginal microbiome to spontaneous prematurity. Disruption of the normal Lactobacillus-dominant vaginal ecosystem may facilitate colonization by pathogenic microorganisms, promote ascending infection, and amplify inflammatory responses within the uterine environment. Such processes have been associated with an increased risk of spontaneous preterm birth and may explain the higher prevalence of positive cultures among singleton pregnancies delivering at earlier gestational ages in our cohort [6]. Furthermore, interactions between vaginal microbiota composition, cervical shortening, and preventive interventions have been shown to influence the risk of preterm birth, highlighting the complex relationship between microbial colonization and cervical remodeling [13].

Experimental evidence further supports the biological plausibility of these findings. Intra-amniotic inflammation has been shown to activate key molecular pathways involved in parturition and may directly induce preterm birth, emphasizing the central role of inflammatory activation in the pathogenesis of prematurity [4].

In contrast, bacterial infection was not significantly associated with gestational age at delivery among twin pregnancies. Although infection has been implicated in some cases of twin preterm birth, twin gestations are exposed to several additional risk factors that may independently promote premature delivery. Uterine overdistension, increased placental mass, altered endocrine signaling, increased cervical strain, and pregnancy-specific complications have all been proposed as important contributors to prematurity in twin pregnancies [7]. Consequently, the relative contribution of bacterial infection may be reduced compared with singleton gestations, where infection-related pathways may play a more dominant role.

The significant interaction identified between gestational type and bacterial infection further supports the hypothesis that the mechanisms underlying preterm birth differ between singleton and twin pregnancies. Although inflammatory pathways contribute to preterm birth across gestational types, twin pregnancies are additionally characterized by uterine overdistension, increased placental mass, altered endocrine signaling, and accelerated cervical remodeling, factors that may exert a greater influence on prematurity than infection alone [2,7]. These observations suggest that culture positivity may be associated with earlier preterm birth in singleton pregnancies. However, because the exposure combined heterogeneous microbiological findings across different sampling sites, organisms, and gestational time points, the present study cannot determine which specific infectious or inflammatory pathways may underlie this association.

### 4.3. Comparison with Previous Studies

The present findings are broadly consistent with previous evidence demonstrating a strong association between bacterial infection and spontaneous preterm birth. Clinical and experimental studies have consistently shown that microbial invasion of the reproductive tract and subsequent inflammatory activation are associated with an increased risk of spontaneous preterm labor and delivery, particularly before 34 weeks of gestation [3,11,12]. Our observation that bacterial infection was significantly associated with delivery before 33 weeks in singleton pregnancies supports these findings and further emphasizes the importance of infection as a contributor to early prematurity.

The association observed in singleton pregnancies is further supported by studies demonstrating links between vaginal microbiome composition and spontaneous preterm birth. Fettweis et al. identified specific vaginal microbial signatures associated with preterm delivery, while Kindinger et al. demonstrated interactions between vaginal microbiota, cervical shortening, and preventive interventions that influence preterm birth risk [6,13]. Together, these findings support the concept that bacterial colonization and host–microbial interactions contribute significantly to the biological processes leading to premature parturition.

By contrast, evidence regarding infection-associated prematurity in twin pregnancies remains limited and less consistent. Previous studies have suggested that risk factors identified in singleton pregnancies may have weaker associations in twin gestations because mechanical and multifactorial pathways contribute substantially to prematurity [2,7]. Our finding that bacterial infection was not significantly associated with gestational age at delivery among twins supports this hypothesis and suggests that infection may play a less prominent role in twin pregnancies than in singleton pregnancies.

Another important finding of the present study was the absence of a significant interaction between bacterial infection and cervical intervention. Neither cervical pessary nor cerclage significantly altered the relationship between bacterial infection and preterm birth. Although cervical interventions may provide mechanical support and reduce the consequences of cervical shortening, they do not directly address infection-related biological mechanisms. Persistent inflammatory activation may continue to promote membrane weakening and cervical remodeling despite mechanical reinforcement of the cervix [8,9,10]. Accordingly, the absence of a statistically significant interaction in the present study should not be interpreted as evidence that cervical interventions are ineffective, but rather that no modifying effect could be demonstrated within the limitations of the present cohort.

The lack of a significant intervention effect is also consistent with contemporary evidence regarding prevention of preterm birth in twin pregnancies. A recent systematic review and network meta-analysis concluded that progesterone, cerclage, and pessary have not consistently demonstrated significant reductions in preterm birth or adverse perinatal outcomes among twin gestations [14]. Together with our findings, these data suggest that the mechanisms responsible for prematurity in twin pregnancies are complex and may not be adequately addressed by a single preventive intervention.

### 4.4. Clinical Implications

The present findings may have important clinical implications. In singleton pregnancies, the presence of a positive bacterial culture may contribute to the overall assessment of preterm birth risk when interpreted alongside established maternal, obstetric, and cervical risk factors. However, the present study does not establish an independent predictive role or support changes in clinical management based on culture positivity alone.

Conversely, the absence of a significant association in twin pregnancies suggests that bacterial culture positivity alone may have limited predictive value for prematurity in this population.

Our findings should not be interpreted as indicating that bacterial infection is unimportant in twin pregnancies. Rather, they suggest that the relative contribution of infection may differ according to gestational type. In twin gestations, preterm birth is a complex multifactorial syndrome in which uterine overdistension, increased placental mass, cervical remodeling, endocrine adaptations, and inflammatory pathways interact. Consequently, the effect of bacterial infection may be less readily detectable than in singleton pregnancies. Furthermore, the relatively modest number of twin pregnancies and the retrospective design may have limited the statistical power to detect smaller associations. These findings should therefore be regarded as hypothesis-generating and require confirmation in larger prospective multicentre cohorts.

The significant interaction observed between gestational type and bacterial infection also emphasizes the importance of individualized risk assessment. Studies that evaluate singleton and twin pregnancies together may underestimate important biological differences between these populations. Future predictive models and preventive strategies should therefore consider gestational type as a potential modifier of infection-associated risk.

## 5. Strengths and Limitations

The present study has several strengths. First, it directly compared singleton and twin pregnancies within the same institutional cohort, allowing assessment of whether bacterial infection exerts differential effects according to gestational type. Second, bacterial infection was defined using documented microbiological culture results rather than clinical suspicion alone, reducing the risk of exposure misclassification. Third, interaction analyses were performed to evaluate both intervention-specific and gestational-type-specific effects, providing a more comprehensive assessment of the relationship between bacterial infection and preterm birth.

Several limitations should also be acknowledged. The retrospective design limits causal inference and introduces the possibility of residual confounding and selection bias. In addition, microbiological cultures were obtained according to routine clinical practice rather than a predefined study protocol, introducing the possibility of confounding by indication. Women with symptoms, cervical changes, or signs of impending preterm labor may have been more likely to undergo microbiological investigation. Therefore, reverse causality cannot be excluded, as clinical features associated with impending preterm birth may have prompted culture sampling rather than culture positivity preceding and contributing to preterm birth.

The study was conducted at a single tertiary referral center, potentially limiting generalizability to other populations. The relatively modest sample size may have reduced statistical power, particularly in subgroup and interaction analyses, resulting in wide confidence intervals for several regression estimates. In addition, information regarding bacterial species, microbial burden, timing of infection, recurrence of infection, and antibiotic treatment was not consistently available. Furthermore, maternal age, parity, previous preterm birth, and antibiotic treatment were not consistently available for all participants and could therefore not be incorporated into the regression models. Their absence may have resulted in residual confounding and limits interpretation of the reported associations as independent effects.

Previous studies have demonstrated that different microorganisms may confer varying levels of risk for preterm birth, and future investigations should incorporate more detailed microbiological characterization. Finally, intervention allocation was based on routine clinical practice rather than randomization, making confounding by indication an unavoidable limitation.

## 6. Conclusions

Ιn this retrospective cohort, bacterial culture positivity was associated with earlier preterm birth in singleton pregnancies in unadjusted analyses, although this association was not confirmed in the multivariable regression analyses. No significant association was observed in twin pregnancies, and cervical intervention type did not significantly modify the relationship between culture positivity and preterm birth.

## Figures and Tables

**Table 1:** Study population and bacterial infection status.

Characteristic	Singleton (n = 70)	Twin (n = 76)
Positive culture	31	33
Negative culture	39	43

**Table 2:** Association between bacterial infection and preterm birth in singleton pregnancies.

Gestational Age	Negative Culture n (%)	Positive Culture n (%)	*p* -Value
34–37 weeks	27 (79.4)	7 (20.6)	
28–33 weeks	12 (33.3)	24 (66.7)	<0.001

**Table 3:** Association between bacterial infection and preterm birth in twin pregnancies.

Gestational Age	Negative Culture n (%)	Positive Culture n (%)	*p* -Value
34–36 weeks	19 (59.4)	13 (40.6)	
29–33 weeks	24 (54.5)	20 (45.5)	0.675

**Table 4:** Logistic regression analyses evaluating the association between bacterial infection, intervention type, and preterm birth.

Variable	OR (Exp(B))	95% CI	*p* -Value
**Singleton pregnancies**			
Bacterial infection	0.375	0.049–2.887	0.346
Intervention	2.250	0.387–13.071	0.366
Infection × Intervention	0.232	0.020–2.642	0.239
**Twin pregnancies**			
Bacterial infection	0.709	0.139–3.619	0.681
Intervention	0.245	0.051–1.169	0.078
Infection × Intervention	1.231	0.161–9.391	0.841
**Combined model**			
Pregnancy type	2.229	0.747–6.651	0.151
Bacterial infection	0.821	0.327–2.060	0.675
Pregnancy type × infection	0.158	0.038–0.655	0.011

## Data Availability

The data supporting the findings of this study are available within the article. Additional data are available from the corresponding author upon reasonable request.
